# Relationships of serum MMP-7 and clinical characteristics in choledochal cyst children

**DOI:** 10.1186/s12893-024-02488-y

**Published:** 2024-06-24

**Authors:** Tong Yin, Suyun Chen, Ruijie Zhou, Wei Liu, Mei Diao, Long Li

**Affiliations:** 1 Capital Institute of Pediatrics-Peking University Teaching Hospital, Beijing, China; 2 Department of General Surgery, Capital Institute of Pediatrics, Beijing, China; 3 Department of Pediatric Urology, Fujian Children’s Hospital, Fujian, China; 4 Research Unit of Minimally Invasive Pediatric Surgery on Diagnosis and Treatment, Chinese Academy of Medical Sciences 2021RU015, Beijing, China; 5 Department of Pediatric Surgery, Tsinghua University Affiliated Beijing Tsinghua Changgung Hospital, Beijing, China

**Keywords:** MMP-7, Choledochal cyst, Liver fibrosis, Perforation, Children.

## Abstract

**Background:**

Matrix metalloproteinase-7 (MMP-7) is associated with biliary injury. This study aimed to evaluate the relationships of serum MMP-7 with clinical characteristics in choledochal cysts (CDC) children.

**Methods:**

Between June 2020 and July 2022, we conducted a prospective study of CDCs who underwent one-stage definitive operation at our center. Serum MMP-7 was measured using an enzyme-linked immunosorbent assay. We evaluated the relationships between serum MMP-7 and age, laboratory tests, imaging examinations, liver fibrosis, MMP-7 expression, and perforation.

**Results:**

A total of 328 CDCs were enrolled in the study, with a median serum MMP-7 of 7.67 ng/mL. Higher serum MMP-7 was correlated with younger age at diagnosis (*p* < 0.001), larger cyst sizes (*p* < 0.001), higher liver fibrosis stages (*p* < 0.001), and higher incidence of perforation (*p <* 0.01). Liver MMP-7 was mainly expressed in intrahepatic and extrahepatic biliary epithelial cells. The area under the receiver operating characteristic curve (AUROC) was 0.630 (*p <* 0.001) for serum MMP-7 in predicting perforation. When serum MMP-7 was combined with γ-glutamyl transferase (GGT), the AUROC increased to 0.706 (*p* < 0.001).

**Conclusions:**

Serum MMP-7 was associated with biliary obstruction in CDCs. Patients with high serum MMP-7 were more likely to have severe liver damage and biliary injury, with higher incidences of liver fibrosis and perforation.

## Backgrounds

Choledochal cyst (CDC) is a congenital anomaly characterized by abnormal dilatation of the biliary tract. First described by Vater [1] in 1723, the etiology of CDC remains unclear. The incidence of CDC ranges from 1:100,000 in Western populations [2] to 1:13,000 in Asian countries [3]. CDC patients are typically diagnosed in childhood, and obstructive jaundice is a common manifestation [4]. Early definitive operation is necessary to prevent severe complications such as liver fibrosis, perforation, and malignancy caused by severe biliary injury and cholestasis.

Matrix metalloproteinase-7 (MMP-7), also known as matrilysin-1, is a member of the metzincin superfamily of zinc-dependent endopeptidases found in the extracellular matrix (ECM) [5]. MMP-7 can degrade important components of the basement membrane and extracellular matrix, including collagen type IV, laminin, entactin, elastin, and fibronectin, leading to basement membrane disruption [6]. In the liver, MMP-7 is produced by glandular epithelial cells, cholangiocytes, and macrophages [7].

To date, a few studies have established an association between serum MMP-7 and biliary injury in biliary atresia (BA) patients [8, 9], and no specific report has studied the performance of serum MMP-7 in CDCs. This study aimed to evaluate the relationships of serum MMP-7 with clinical characteristics in CDC children.

## Methods

### Patients and parameters

In this study, CDC patients who underwent one-stage definitive operation at Children’s Hospital Capital Institute of Pediatrics between June 2020 and July 2022 were enrolled. Preoperative abdominal ultrasonography, computed tomography (CT), laboratory tests, and intraoperative cholangiography were conducted routinely, which confirmed the diagnosis.

Enrolled CDC patients were divided into 2 groups: the low-level of serum MMP-7 group (group 1) consisted of serum MMP-7 values ranging from 0 to 80 percentiles, and the high-level of serum MMP-7 group (group 2) consisted of serum MMP-7 values ranging from 80 to 100 percentiles.

Patient characteristics, laboratory tests, and imaging examinations were extracted from the electronic medical record system. For prenatally diagnosed CDCs, the age at diagnosis was defined as a negative number representing the period from the time of prenatal diagnosis to the date of birth. Laboratory tests included serum alanine aminotransferase (ALT), aspartate aminotransferase (AST), total bilirubin (TB), direct bilirubin (DB), alkaline phosphatase (ALP), γ-glutamyl transferase (GGT), total bile acids (TBA), and amylase (AMY). Complete obstruction of the distal common bile duct is a severe subtype of choledochal cyst characterized by the inability of the contrast medium to enter the duodenum through the common bile duct. To reduce the impact of age, a relative cyst size index was adopted for the major papilla lying always near the second lumbar vertebra (L2), we measured the diameter/length of common bile duct and divided it by the height of the second lumbar vertebra, i.e., relative cyst diameter = diameter of the cyst/height of L2. Data were assessed for each patient [10]. The papilla of Vater located at the distal end of the descending duodenum was recognized as ectopic, whereas the papilla of Vater located at the descending duodenum was considered normal [10, 11].

### Serum MMP-7, liver fibrosis and tissue MMP-7 expression measurement

Serum samples were obtained from each patient before surgery, stored at -80 ℃, and used to detect serum MMP-7. Serum MMP-7 concentration was measured using an enzyme-linked immunosorbent assay (ELISA) according to the manufacturer’s protocol (Quantikine, R&D Systems, Inc., Minneapolis, USA).

Liver biopsies were reviewed by two experienced liver pathologists who were blinded to the clinical characteristics of the study subjects. Liver fibrosis stages were classified according to the Ludwig staging system [12]. Ludwig fibrosis stage 0–2 was defined as the non-advanced fibrosis strata group, and Ludwig fibrosis stage 3–4 was defined as advanced fibrosis.

Immunohistochemistry was performed on tissue sections from paraffin-embedded liver biopsies to detect tissue MMP-7 expression using anti-MMP-7 antibody (Abcam, Cambridge, United Kingdom) and ductular proliferation using anti-cytokeratin-19 (CK-19) antibody (Abcam, Cambridge, United Kingdom). Cells containing yellow to brown cytoplasm or membrane were considered positive for tissue MMP-7 expression. Staining was evaluated at ×400 magnification in a minimum of three areas and categorized per specimen based on the scores as follows: no positive cells, 0 points; < 33% positive cells, 1 point; 33 ~ 67% positive cells, 2 points; and positive cells > 67%, 3 points. Specimens were also scored based on the staining intensity as follows: negative, 0 points; pale yellow, 1 point; medium yellow, 2 points; and pale brown, 3 points. The highest staining intensity was rated in the same area, and a total score was obtained by multiplying the two above-mentioned values [13, 14].

### Ethics

This study was carried out in accordance with The Code of Ethics of the World Medical Association (Declaration of Helsinki). The study was approved by Ethical Committee of Capital Institute of Pediatrics (shell2022047), and informed consent was obtained from all subjects and their legal guardian(s)/parents.

### Statistical analysis

Statistical analysis was performed using SPSS version 23.0 (IBM, Armonk, NY, USA). Normally distributed continuous variables were expressed as mean ± SD and analyzed using Student’s t-test or one-way ANOVA. Non-normally distributed variables were expressed as interquartile range and analyzed using the Mann-Whitney U test or Kruskal-Wallis test. Categorical variables described as proportions were compared by χ2 test or Fisher’s exact test. Spearman’s correlation was used to analyze the correlation between different parameters. The diagnostic accuracy was assessed using the area under the receiver operating characteristic (AUROC) curves generated by GraphPad Prism version 8.0 (GraphPad Software, California, USA). A *p*-value of less than 0.05 was considered statistically significant.

## Results

### Patient characteristics

From June 2020 to July 2022, a total of 328 CDC patients were successfully enrolled in this study: 263 patients in group 1 and 65 patients in group 2. In all CDCs, the median serum MMP-7 level was 7.67 (5.26, 13.52) ng/mL, with 6.48 (4.79, 9.40) ng/mL in group 1 and 28.28 (22.59, 38.61) ng/mL in group 2.

No significant difference was noted in the male-female ratio (68/195 vs. 11/54, *p* = 0.147, Table 1) or prenatally diagnosis (41.83% vs. 55.38%, *p* = 0.052). However, the age at diagnosis and age at surgery were significantly younger in group 2 (14.30 vs. -3.27 months, *p* < 0.01; and 24.73 vs. 2.00 months, *p* < 0.001, respectively). Cyst sizes were compared between the two groups, and the cyst diameter and cyst lengths were larger in group 2 (26.14 vs. 35.03 mm, *p* < 0.001; and 50.72 vs. 60.69 mm, *p* < 0.001, respectively). Moreover, the incidences of the complete obstruction of the distal common bile duct, advanced fibrosis, and perforation were significantly higher in group 2 (9.13% vs. 38.46%, *p* < 0.001; 8.57% vs. 22.03%, *p* < 0.01; and 8.75% vs. 26.15%, *p* < 0.001, respectively).

**Table 1:** Patient characteristics and laboratory tests of two groups

Features	Group 1 (*n* = 263)	Group 2 (*n* = 65)	*p*
Serum MMP-7 (ng/mL)	6.48 (4.79, 9.40)	28.28 (22.59, 38.61)	-
Male/female	68/195	11/54	0.147
Prenatally diagnosed, n (%)	110 (41.83)	36 (55.38)	0.052
Age at diagnosis (months)	14.30 (-3.50, 31.27)	-3.27 (-4.20, 22.97)	< 0.01
Age at surgery (months)	24.73 (3.43, 50.10)	2.00 (0.53, 33.28)	< 0.001
Cyst diameter (mm)	26.14 ± 13.63	35.03 ± 17.75	< 0.001
Cyst length (mm)	50.72 ± 20.16	60.69 ± 25.32	< 0.001
Complete obstruction of the distal common bile duct, n (%)	24 (9.13)	25 (38.46)	< 0.001
Advanced fibrosis, n (%)	18 (8.57)	13 (22.03)	< 0.01
Perforation, n (%)	23 (8.75)	17 (26.15)	< 0.001
ALT (U/L)	20.70 (12.70, 41.40)	48.50 (13.65, 116.90)	< 0.01
AST (U/L)	32.40 (26.00, 43.40)	49.70 (30.60, 97.45)	< 0.001
TB (µmol/L)	9.20 (6.40, 18.60)	129.00 (31.40, 186.80)	< 0.001
DB (µmol/L)	2.50 (1.70, 5.60)	16.90 (10.75, 34.15)	< 0.001
ALP (U/L)	238.00 (186.00, 300.00)	308.00 (212.50, 442.00)	< 0.001
GGT (U/L)	61.00 (20.00, 193.50)	463.20 (197.35, 643.50)	< 0.001
TBA (µmol/L)	4.30 (2.10, 7.10)	13.20 (4.00, 42.60)	< 0.001
AMY (U/L)	48.00 (15.00, 84.00)	10.00 (6.00, 52.50)	< 0.001

### Relationships of serum MMP-7 and age

**Fig. 1:**
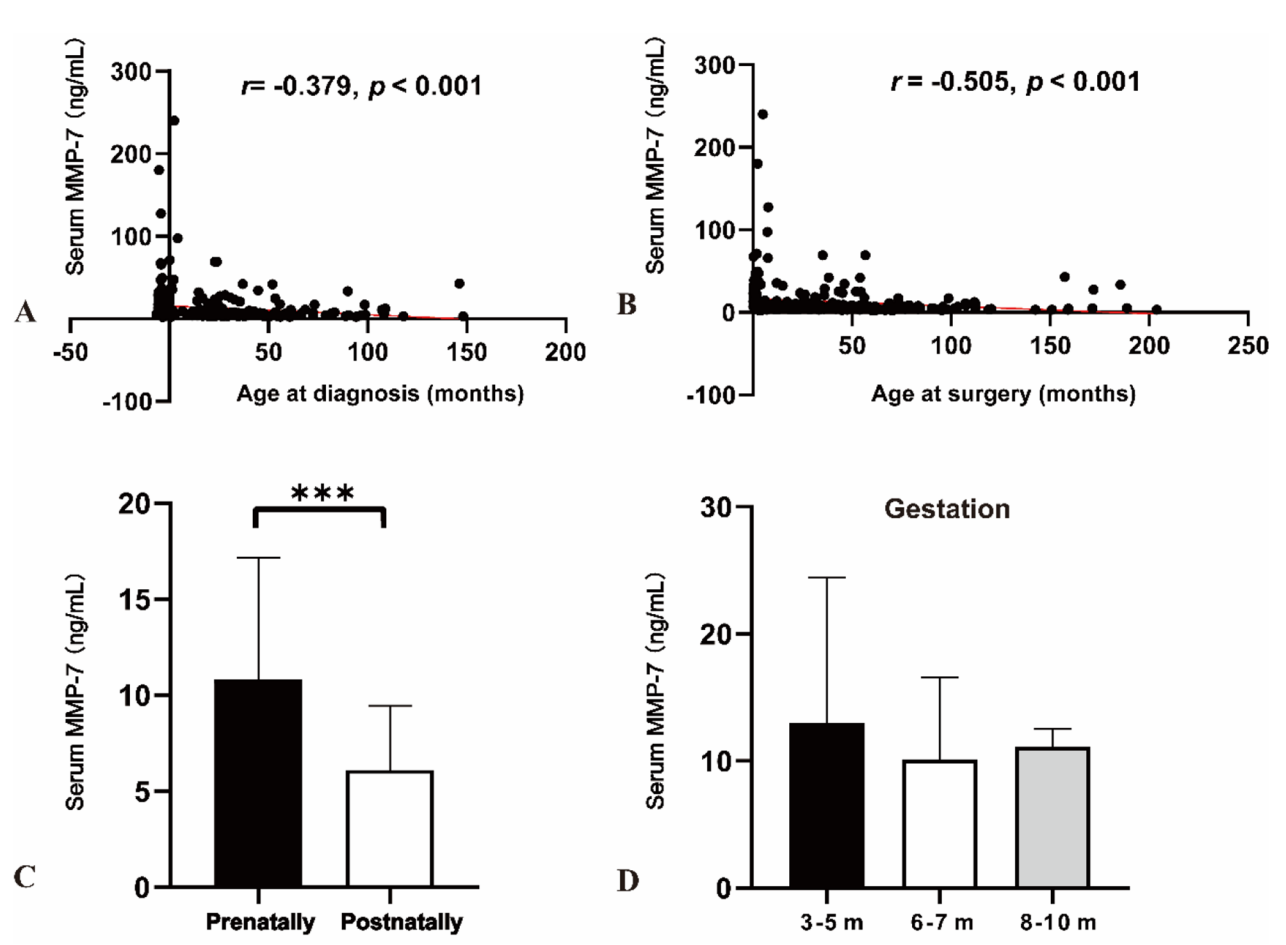
Relationship of serum MMP-7 and age: (**A**) correlation between serum MMP-7 with age at diagnosis; (**B**) correlation between serum MMP-7 with age at surgery; (**C**) comparison of serum MMP-7 levels in prenatally diagnosed group and postnatally diagnosed group; and (**D**) comparison of serum MMP-7 levels at different gestational ages. ***: *p <* 0.001

As shown in Fig. 1, we analyzed the relationship between serum MMP-7 and age parameters. We found that serum MMP-7 was negatively correlated with age at diagnosis (*r* = -0.379, *p* < 0.001) and age at surgery (*r* = -0.505, *p* < 0.001). Based on age at diagnosis, we divided patients into two groups: the prenatally diagnosed group (*n* = 146, 44.51%) and the postnatally diagnosed group (*n* = 182, 55.49%). Analysis revealed that CDC patients in the prenatally diagnosed group had significantly higher serum MMP-7 than those in the postnatally diagnosed group (10.84 vs. 6.10 ng/mL, *p* < 0.001).

Furthermore, when comparing patients diagnosed at different gestational ages, we found that serum MMP-7 was slightly higher in patients diagnosed in early pregnancy (3 to 5 months) than those diagnosed in late pregnancy (6 to 7 months and 8 to 10 months). However, the difference was not statistically significant (12.97 vs. 10.15 vs. 11.11 ng/mL, *p* = 0.218).

### Relationships of serum MMP-7 and laboratory tests

**Fig. 2:**
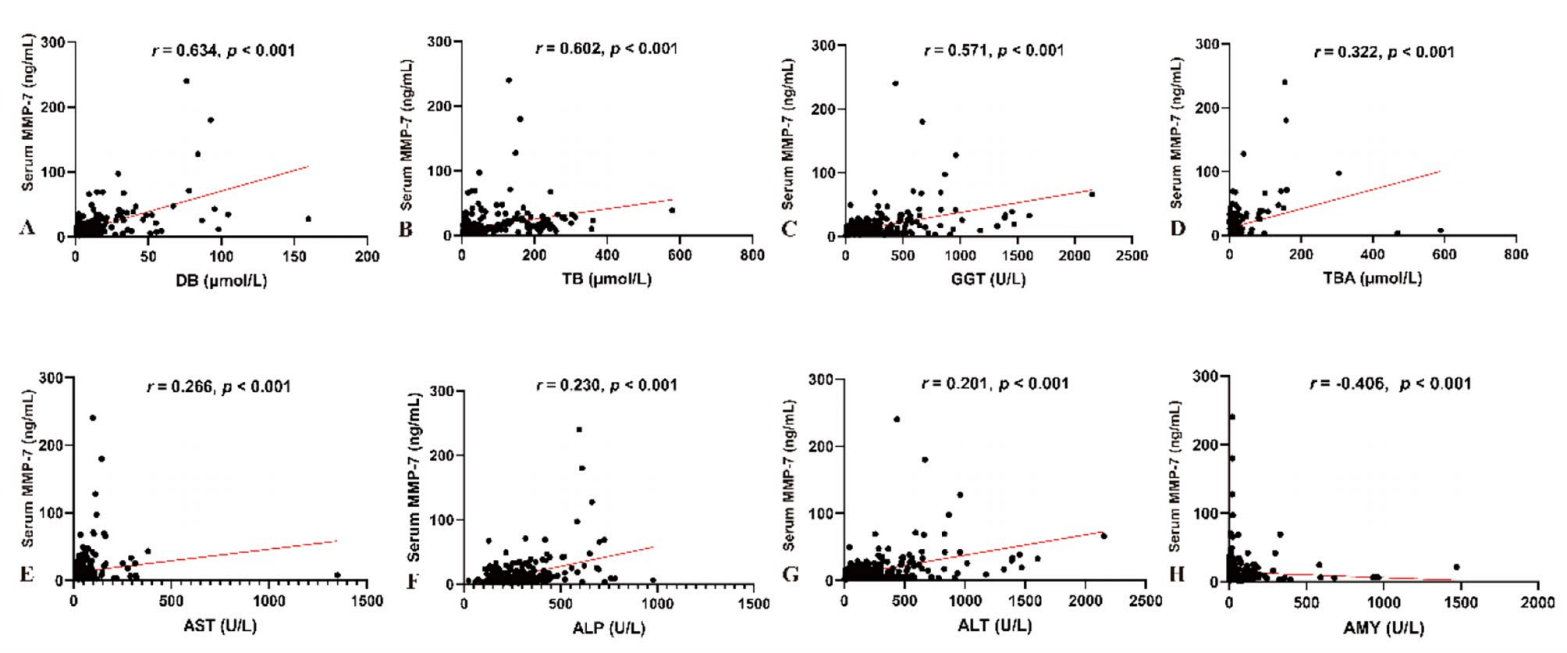
Correlations between serum MMP-7 with laboratory tests: serum MMP‐7 with DB (**A**), TB (**B**), GGT (**C**), TBA (**D**), AST (**E**), ALP (**F**), ALT (**G**), and AMY (**H**)

The relationships between serum MMP-7 and laboratory tests were investigated, revealing a strong correlation between serum MMP-7 and liver damage and biliary injury. As shown in Fig. 2, serum MMP-7 was positively correlated with DB (*r* = 0.634, *p* < 0.001), TB (*r* = 0.602, *p* < 0.001), GGT (*r* = 0.571, *p* < 0.001), TBA (*r* = 0.322, *p* < 0.001), AST (*r* = 0.266, *p* < 0.001), ALP (*r* = 0.230, *p* < 0.001), and ALT (*r =* 0.201, *p* < 0.01), and negatively correlated with AMY (*r* = -0.406, *p* < 0.001). Furthermore, preoperative laboratory tests were compared between the two groups, and all laboratory tests were significantly more deranged in group 2 (ALT: *p* < 0.01; AST: *p* < 0.001; TB: *p* < 0.001; DB: *p* < 0.001; ALP: *p* < 0.001; GGT: *p* < 0.001; TBA: *p* < 0.001; and AMY: *p* < 0.001, respectively, Table 1).

### Relationships of serum MMP-7 and imaging examinations

**Fig. 3:**
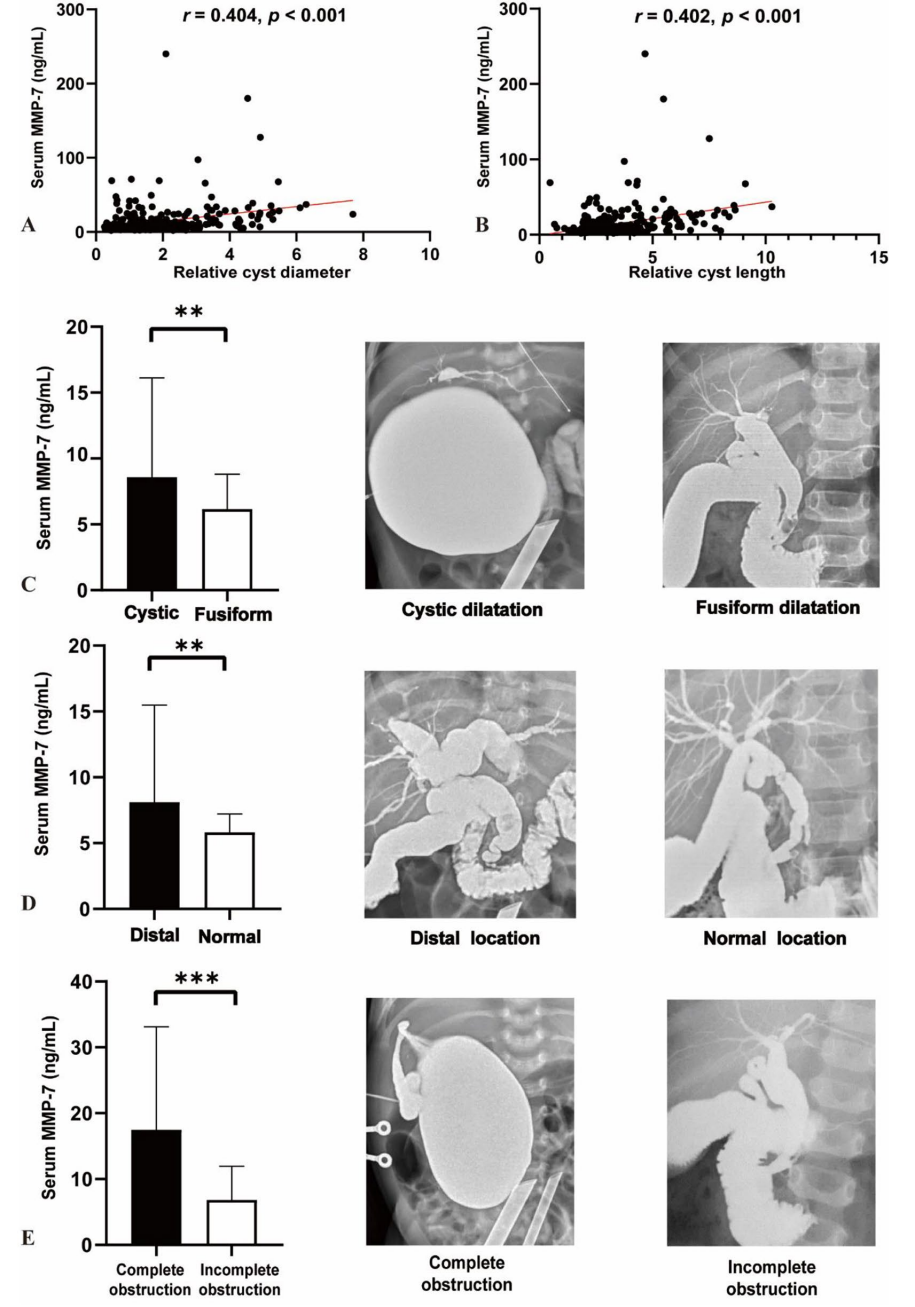
Relationships of serum MMP-7 and imaging examinations: (**A**) correlation between serum MMP-7 with relative cyst diameter; (**B**) correlation between serum MMP-7 with relative cyst length; (**C**) comparison of serum MMP-7 in cystic and fusiform dilatation groups; (**D**) comparison of serum MMP-7 in ectopic distal and normal location of the papilla of Vater groups; (**E**) comparison of serum MMP-7 in complete and incomplete obstruction of the distal common bile duct groups; **: *p <* 0.01; ***: *p <* 0.001

After adjusting for age, serum MMP-7 demonstrated a significant positive correlation with both the mean relative cyst diameter (1.85 ± 1.27, *r* = 0.404, *p* < 0.001) and mean relative cyst length (3.28 ± 1.58, *r* = 0.402, *p* < 0.001, Fig. 3) in all CDC patients.

Cystic dilatation was confirmed in 259 (78.95%) CDCs while fusiform dilatation was present in 69 (21.04%) patients. Notably, serum MMP-7 were significantly higher in patients with cystic dilatation compared to those with fusiform dilatation (8.58 vs. 6.16 ng/mL, *p* < 0.01). The ectopic distal location of the papilla of Vater was observed in a high proportion of patients (88.51%, 285/322). Those with CDC in this location had higher serum levels of MMP-7 compared to patients with a normal location of the papilla of Vater (8.10 vs. 5.83 ng/mL, *p* < 0.01). Additionally, patients with complete obstruction group exhibited significantly elevated serum MMP-7 compared to those with incomplete obstruction (17.47 vs. 6.82 ng/mL, *p* < 0.001).

### Relationships of serum MMP-7, liver fibrosis and MMP-7 expression

**Fig. 4:**
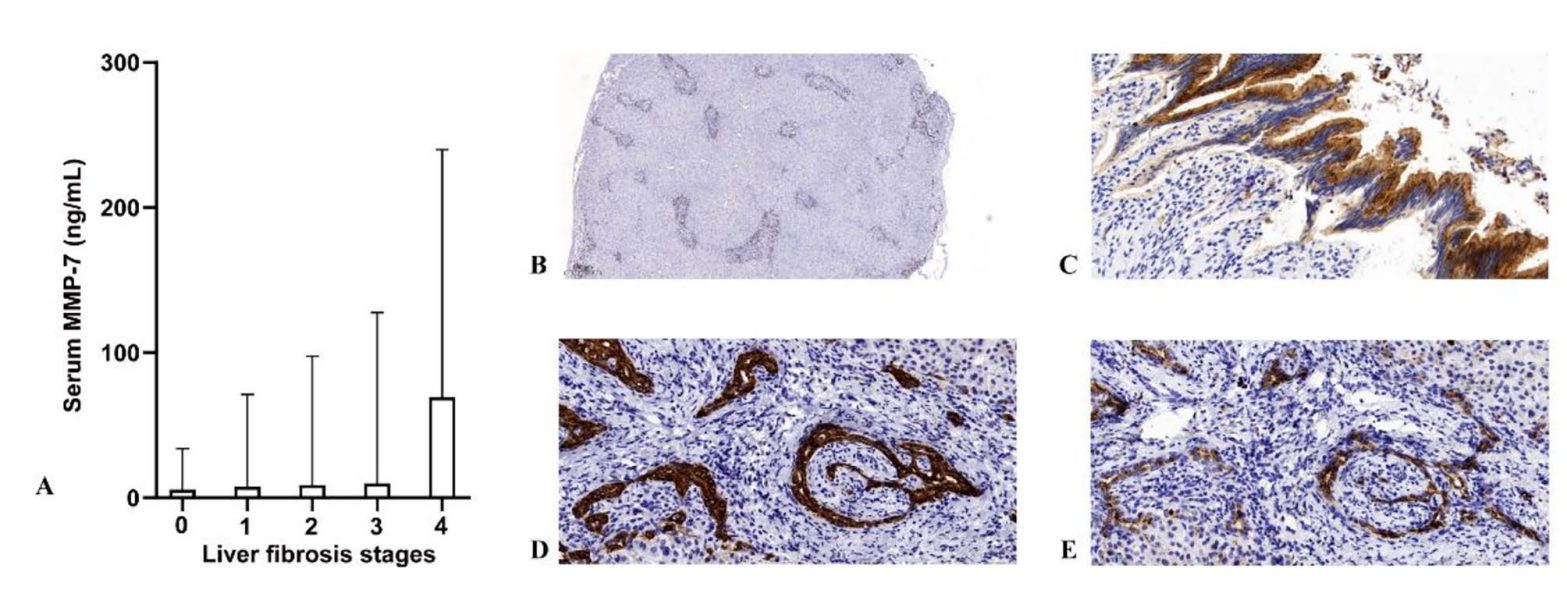
Relationships of serum MMP-7, liver fibrosis, and MMP-7 expression: (**A**) serum MMP-7 in different fibrosis stages; (**B**) liver MMP-7 immunostaining (×40); (**C**) extrahepatic biliary duct MMP-7 immunostaining (×400); (**D**) liver MMP-7 immunostaining (×400); (**E**) liver CK-19 immunostaining, showing the same area of (D)

A total of 269 liver biopsy results were analyzed, which included 41 cases of stage 0 fibrosis, 103 cases of stage I, 94 cases of stage II, 26 cases of stage III, and 5 cases of stage IV. Statistical analysis revealed a significant positive correlation between serum MMP-7 and fibrosis stage (stage 0 vs. stage I vs. stage II vs. stage III vs. stage IV: 5.71 vs. 7.71 vs. 8.67 vs. 9.89 vs. 69.13 ng/mL: *p <* 0.001, Fig. 4).

Limited immunostaining showed that MMP-7 was predominantly expressed in intrahepatic and extrahepatic biliary epithelial cells (Fig. 4B and C). A common expression pattern was identified between MMP-7 and CK-19 in liver tissues (Fig. 4D and E). In correlative analyses of this cohort, a positive association was observed between serum MMP-7 and MMP-7 expression in the liver (*p* < 0.01) and in the extrahepatic biliary ducts (*p* < 0.05).

### Diagnostic value of serum MMP-7 for perforation

**Fig. 5:**
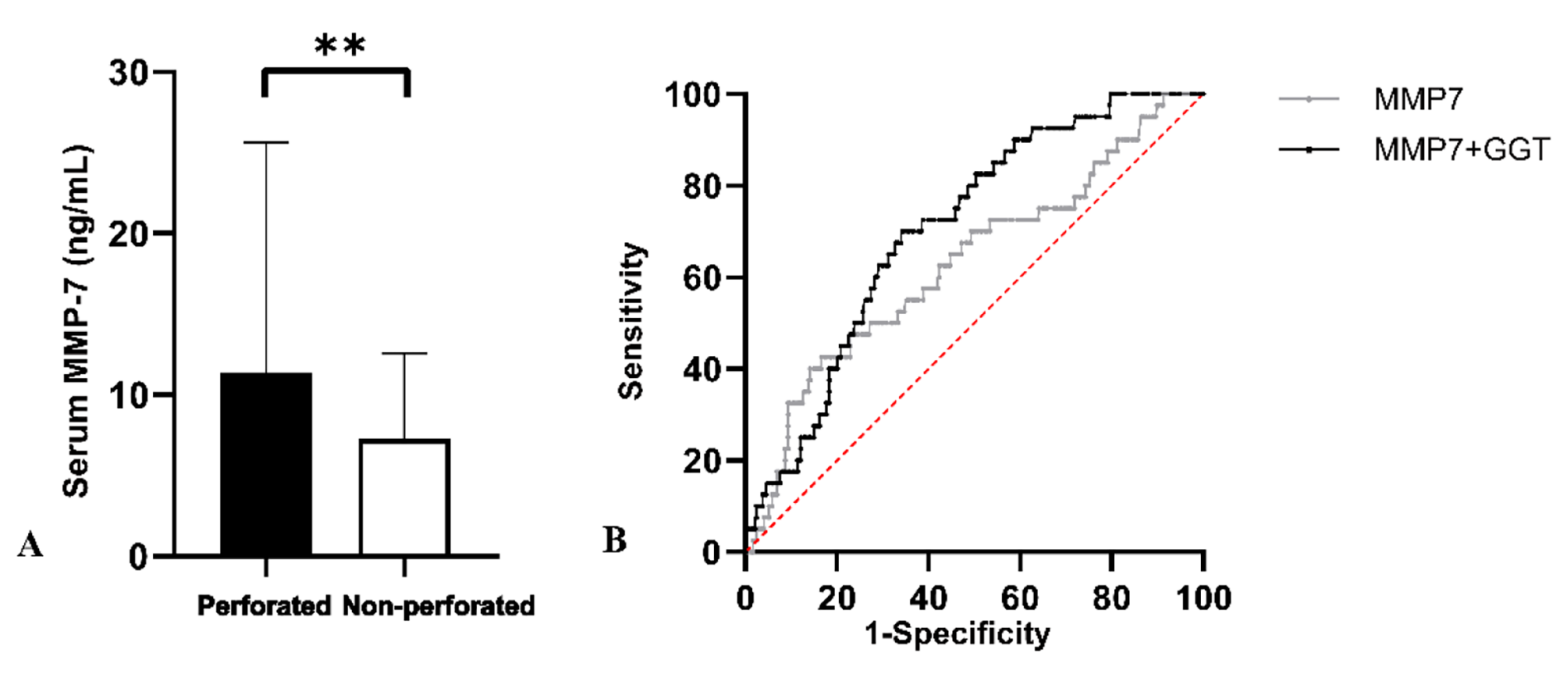
Diagnostic value of serum MMP-7 for perforation diagnosis: (**A**): serum MMP-7 in perforated and non-perforated CDCs; (**B**) the ROC curves in diagnosis of perforation for CDCs.

Perforation was intraoperatively confirmed in 40 (12.20%) individuals, who had significantly higher serum MMP-7 compared to non-perforated patients (11.39 vs. 7.26 ng/mL, *p <* 0.01, Fig. 5).

To assess the diagnostic performance of serum MMP-7 in perforation, the AUROC curve was calculated. For detecting perforation in CDCs, the optimal cutoff value for serum MMP-7 was determined to be 17.15 ng/mL, with an AUROC, sensitivity, and specificity of 0.630 (95% CI: 0.533 ~ 0.723, *p <* 0.001), 42.50%, and 83.33%, respectively. When serum MMP-7 was combined with GGT, the AUROC was 0.706 (95% CI: 0.631 ~ 0.782, *p* < 0.001), with sensitivity and specificity values of 70.00% and 65.97%, respectively.

## Discussion

In this study, a significant correlation was observed between serum MMP-7 and biliary obstruction in CDCs. Patients with elevated serum MMP-7 were more likely to experience severe obstruction of the distal end of the common bile duct, as evidenced by abnormal laboratory tests (higher levels of GGT, TB, and DB), larger cyst sizes, and higher incidence of perforation. Besides, the CDCs with higher levels of serum MMP-7 were more likely to suffer liver damage, convinced by the higher levels of ALT and AST, and a higher rate of liver fibrosis. In CDC patients, biliary obstruction might be the major factor of liver damage and further liver fibrosis [15, 16]. Therefore, it is postulated that the impact of serum MMP-7 on liver damage may be mediated through biliary obstruction.

The decision to use the 80th percentile as the threshold for dividing the two groups was based on several important considerations. First, data analysis revealed that patients with serum MMP-7 values in the 80th to 100th percentiles exhibited a significantly higher rate of complete obstruction, advanced fibrosis, and perforations compared to those in the 0th to 80th percentiles range. Second, there were notable similarities in biliary injuries and liver damage between CDC patients in group 2 and BA patients in terms of indistinguishable serum MMP-7, GGT, and DB levels, as well as liver fibrosis stages (unreported data). This finding is particularly concerning, especially given that the majority of CDC patients were not in the acute phase at the time of surgery, suggesting that actual outcomes could potentially be more severe than those observed in this study, further stressing the severity of CDC and necessity of prompt definitive operation.

As a member of the group of matrix metalloproteinase family, MMP-7 was implicated in ECM turnover via proteolytically cleavage and inflammation [6]. In this study, MMP-7 expression was predominantly identified in intrahepatic and extrahepatic biliary epithelial cells, with a positive correlation observed between serum MMP-7 levels and MMP-7 expression, indicating the release of MMP-7 to the serum triggered by the biliary epithelial injury, consistent to the study by Lertudomphonwanit [8].

Immunohistochemistry in liver sections in current study revealed predominant MMP-7 expression in intrahepatic biliary epithelial cells, which had a similar expression pattern to CK-19. This observation may be attributed to the obstruction of the distal common bile duct causing the intrahepatic biliary epithelial cells damage, leading to fibroblastic proliferation, scar tissue formation, bile excretion decreases, and cholestasis, further resulting in the bile duct hyperplasia, accompanied by MMP-7 protein expression.

According to Wu’s report, the serum MMP-7 was lower in BA patients who underwent a cholestatic workup at a younger age [17]. However, our cross-sectional research on age at diagnosis revealed a negative correlation between serum MMP-7 levels and increasing age in CDC patients. These opposite results could be explained by the relationships between serum MMP-7 and biliary obstruction as well as liver damage. In BA patients, serum MMP-7 might elevate with age, as the liver suffers more damage. Conversely, in CDC patients, younger individuals may exhibit more severe biliary obstruction and liver damage, especially in prenatally diagnosed CDCs [4, 18]. Therefore, prenatally diagnosed patients were more likely to experience liver fibrosis and perforation and had higher levels of serum MMP-7 than postnatally diagnosed patients in our study, which further emphasized the importance of prompt surgery. Moreover, serum MMP-7 was slightly higher in patients diagnosed at early pregnancy (3–5 months) than in those diagnosed later, suggesting severe hepatic tissue remodeling during this period of gestation.

Complete obstruction of the distal common bile duct is a severe subtype of choledochal cyst characterized by the inability of contrast medium to enter the duodenum through the common bile duct, and few clinical studies have reported this subtype [19–21]. Our findings revealed higher serum MMP-7 in the complete obstruction group compared to the incomplete obstruction group of CDCs. In our series, most patients with complete obstruction were diagnosed prenatally (28/49, 57.14%), and had a pretty younger age than those CDCs without complete obstruction (age at diagnosis: -3.27 vs. 13.60 months, *p* < 0.01). Complete obstruction can elevate intraluminal pressure, further contribute to liver damage and biliary injury, and increase the incidence of liver fibrosis and perforation. Animal experiments also showed that ligation of the distal end of the common bile duct in newborn lambs can lead to the weakening of the bile duct wall, massive dilatation of the extrahepatic biliary duct, and liver fibrosis [22, 23].

The papilla of Vater represents the orifice of the hepatic diverticulum of the early embryo [24]. An ectopic distal location of the hepatic diverticulum could cause stretching and weakness of the common bile duct and common channel, leading to distal stenosis and a longer common channel, ultimately resulting in choledochal cysts [10]. Our previous report showed that patients with a more distal location of the papilla of Vater had larger cysts and more severe clinical features [11]. This study further demonstrated a correlation between the location of the papilla and serum MMP-7, with more distal locations associated with higher levels of MMP-7.

Previous studies had reported a positive correlation between MMP-7 and other fibrotic diseases, including renal fibrosis and pulmonary fibrosis [6, 25], but there was no literature has studied the relationship between serum MMP-7 and liver fibrosis in CDCs. Our study confirmed a positive correlation between serum MMP-7 and liver fibrosis stages, which might have resulted from biliary obstruction as reported [15, 16]. These findings also suggest that serum MMP-7 might be a helpful parameter to predict liver fibrosis preoperatively and to follow up retrogression in different recovery stages.

Perforation is a severe complication of CDCs that can lead to life-threatening sepsis, infectious shock, and even mortality if not treated promptly [26]. Distal obstruction of the common bile duct could increase the intraluminal pressure and contribute to perforation [27]. Our study found that serum MMP-7 was significantly higher in patients with perforated CDCs. To detect perforation at an early stage, we analyzed the diagnostic performance of MMP-7 in differentiating perforated from non-perforated cases, identifying an optimal cutoff value of 17.15 ng/mL suggestive of perforation. Combining serum MMP-7 and GGT increased predictive accuracy, with an AUROC of 0.706, indicating the valuable performance of GGT in choledochal cyst perforation [18].

In this prospective study, the serum samples were collected after the start of the study instead of using the serum stored in the refrigerator for a long time to mitigate potential degradation. Besides, the serum was collected before medical treatment to reduce the impact of drugs and operation, and stored in a refrigerator (-80 °C) to avoid repeated freezing and thawing. ELISA procedures were conducted within a week of collection to reduce degradation, and other laboratory test results were detected on the same day. We also used the same ELISA kits as previously reported in other diseases, including BA [9, 28, 29], to address potential issues with value variation among different kits.

However, this study has several limitations, including its single-center nature, lack of sufficient histological expression data, and absence of postoperative follow-up. Therefore, a long-term and multicenter study is needed in the future.

In conclusion, our study demonstrates a strong correlation between serum MMP-7 and biliary obstruction in patients with CDC. Higher serum MMP-7 levels were found to be associated with more severe liver damage and biliary injury, and a higher incidence of liver fibrosis, and perforation.

## Data Availability

The datasets generated and/or analyzed during the current study are not publicly available due to the protection of personal privacy but are available from the corresponding author on reasonable request.

## Abbreviations

MMP-7: Matrix metalloproteinase-7
CDC: Choledochal cysts
AUROC: Area under the receiver operating characteristic curve
GGT: γ-glutamyl transferase
ECM: Extracellular matrix
BA: Biliary atresia
CT: Computed tomography
ALT: Alanine aminotransferase
AST: Aspartate aminotransferase
TB: Total bilirubin
DB: Direct bilirubin
ALP: Alkaline phosphatase
TBA: Total bile acids
AMY: Amylase
L2: the second lumbar vertebra
ELISA: Enzyme-linked immunosorbent assay
CK-19: Cytokeratin-19
